# Willingness to accept malaria vaccine among caregivers of under-5 children in Ibadan North Local Government Area, Nigeria

**DOI:** 10.5281/zenodo.10870005

**Published:** 2015-03-23

**Authors:** Beliretu I Abdulkadir, Ikeoluwapo O Ajayi

**Affiliations:** 1 Department of Epidemiology and Medical Statistics, Faculty of Public Health, College of Medicine, University of Ibadan, Oyo State, Nigeria

## Abstract

**Background:**

Malaria vaccine is a potentially effective addition to the armamentarium for malaria control. The candidate RTS,S malaria vaccine has undergone phase III clinical trials and WHO has indicated that a policy recommendation is possible in 2015. Given the delays with adoption of other novel interventions including vaccines, there is a need to ensure that all elements that will inform the decision to adopt a malaria vaccine, including community willingness, will be available on time. This study was carried out to assess the willingness of caregivers of under-5 children to accept a malaria vaccine once available and recommended for use.

**Materials and Methods:**

427 consenting caregivers, selected using a cluster sampling technique, from five communities in Ibadan, Nigeria, participated in a questionnaire. In-depth interviews (IDIs) were conducted among 47 key community members. Data were analysed using descriptive statistics, Chi-square and logistic regression at *p*≤0.05. Thematic content analysis was used to analyse the transcribed IDI data.

**Results:**

The mean age of survey respondents was 29.8±5.8 years. Only 20.1% of the respondents had ever heard of malaria vaccine; 87.0% showed willingness to accept a malaria vaccine. Reasons stated for not willing included ‘husband did not want immunisation’ (73.6%), ‘felt it might be expensive’ (47.2%) and ‘felt it might paralyse children’ (24.5%). Nearly half (48.7%) of the respondents said that if vaccine is not given orally like polio vaccine it might not be accepted. Influence of community health workers was found to predict willingness to accept a malaria vaccine (OR: 0.316, 95% CI: 0.142-0.705). IDI participants were favourably disposed to introduction of a vaccine against malaria, although they had concerns about the formulation of the vaccine and possible adverse events.

**Conclusion:**

Well-designed communication strategies implemented prior to the introduction of a malaria vaccine would be essential to foster a supportive environment for eventual adoption and acceptance thereof.

## 1 Introduction

Malaria control has received a commendable increase in political and financial commitments in the past decade. There has been scale-up of vector control interventions, diagnostic testing and treatment with artemisinin-based combination therapies (ACTs) in many endemic countries, and this cumulatively has resulted in 3.3 million lives saved from the disease [1]. According to the World Health Organization (WHO), malaria mortality rates were reduced by about 42% globally and by 49% in the WHO African Region between 2000 and 2012. During the same period, malaria incidence rates were reduced by 25% globally, and by 31% in the WHO African Region [1]. In Nigeria, malaria remains an important cause of morbidity and mortality. An estimated 97% of the country’s approximate population of 160 million residents are at risk of malaria, with children under the age of five and pregnant women being the most vulnerable. Death from malaria in Nigeria accounted for 32% of the global estimate of malaria deaths in 2010 [2]. There is no official report of reduction in the malaria incidence and mortality in the country. However, there are speculations that malaria incidence is on the decrease.

In line with WHO recommendations, and following evidence of effectiveness of malaria control interventions from earlier studies, the National Malaria Control Programme in Nigeria has scaled up these interventions. The progress made so far included an increase in the distribution of long-lasting insecticide-treated nets (LLINs), policy review and strategic plan development, scaling-up of access to ACTs and rapid diagnostic tests (RDT) for malaria, as well as the expansion of the plan for vector management under the Integrated Testing, Treatment and Larval control programme (ITTLC) [3]. However, these activities have been faced with some challenges. Although appreciable successes have been recorded for universal coverage of LLINs for vector control, the actual use is suboptimal. In addition, scaling up of intermittent preventive treatment during pregnancy (IPTp) and indoor residual spraying (IRS) continues to be a challenge because of poor compliance and coverage, respectively. Distribution of ACTs in the treatment of uncomplicated malaria is faced with a disjointed commodity supply system, poor compliance and inadequate backup with diagnostics for appropriate use [4]. Some of these challenges, which have slowed down the achievement of the various control targets, could be attributed to poor social marketing of the commodities and awareness creation. Thus, the need to forestall such inadequacies is required in order to achieve successful introduction of a malaria vaccine into the country if and when it becomes available and recommended for use.

Another important factor which has brought the remarkable recent achievements in the control of malaria under serious threat is the emergence of resistance to artemisinin, the core compound in ACTs, by *Plasmodium* species and resistance of the *Anopheles* mosquito to pyrethroid insecticides. Whereas global strategies to tackle this challenge have been proffered by WHO there is need for additional effective preventive measures to these complement efforts. An effective vaccine against malaria has long been envisaged as a valuable addition to the available tools for malaria control [1], and this led to the launch of research and development of the vaccine with the support of WHO and malaria vaccine funders.

For many years and especially in the last few decades, organisations like PATH-Malaria Vaccine Initiative, the Bill and Melinda Gates Foundation and WHO have supported companies such as GlaxoSmithKline with the development of a vaccine against malaria. Many potential anti-malarial vaccines have been formulated and evaluated in clinical trials, including the GlaxoSmithKline vaccine candidate RTS,S, which has progressed to Phase 3 clinical trials. The WHO has indicated that RTS,S, following satisfactory effectiveness in the recent Phase 3 clinical trials, will be recommended, and it is foreseen that by 2015/6 the vaccine may be available for use [5].

One major issue in the use of a new intervention is the willingness of the target population to accept and use it. Misinformation within communities or poorly handled information by decision makers could result in an outright lack of support for an intervention after it has been introduced [6]. A distrustful climate between communities and immunisation programmes can contribute to growing pools of non-immunised or partially immunised children [6-8]. Stanton [9] pointed to the key role that trusted sources or opinion leaders and health professionals can play in fostering acceptance for vaccination.

PATH-MVI stated that, experience has shown that the development of an innovative health intervention does not necessarily mean that it will be adopted, delivered, accepted and used immediately in a way that will make significant impact on people’s health [10]. There are several, interrelated technical, individual, political, financial and social issues that influence the adoption and implementation of new health interventions. Late attention to these issues is likely to result in a delayed policy decision regarding a health technology or in a decision being taken without enough information to support it and facilitate its use [10].

This study set out to assess the willingness to accept a malaria vaccine and the correlates among individuals who influence decisions on childhood vaccination. It is expected that the findings will motivate the relevant stakeholders to consider preparing early for a possible adoption of a malaria vaccine with proven effectiveness. In addition, it will provide evidence to focus on specific areas identified to be potential mitigating factors to communities’ willingness to accept the vaccine.

## 2 Materials and Methods

### 2.1 Study Design

A cross-sectional household survey and in-depth interviews (IDIs) were used in this study.

### 2.2 Study population and site

Caregivers of children less than 5 years old and opinion leaders in five communities in Ibadan North Local Government Area (IBNLGA) were interviewed. IBNLGA is one of the most densely populated areas in Oyo State with a total population of 306,795 inhabitants (2006 census data). The major occupation of the people is trading, followed by artisans, while others are in public service and/or engage in small-scale manufacturing.

### 2.3 Sample size determination

Sample size determination for this study was calculated using the Leisle−Kish formula for sample size determination for single proportion. As the authors could not obtain literature that reported similar studies in this environment, prevalence was estimated to be 50%. Thus, sample size was calculated to be 385 using standard normal deviate of 1.96 at 95% confidence interval and precision of 5%. When a possible 10% non-response was considered, the minimum sample size needed for the study became 427.

A two-stage cluster sampling technique was used to select participants for the quantitative study. Five wards were randomly sampled from the 12 wards in IBNLGA. Information on the number of communities in the selected wards was obtained from the local government secretariat. One community was randomly selected from each of the selected wards. The selected communities formed a cluster for the study. The research covered every consenting household in these communities until the sample size was achieved. In households with more than one eligible person, balloting was used to pick one eligible participant.

The IDIs were carried out among purposively selected key members of the communities, including opinion leaders. These are people who influence whether a child is immunised, based on their experience, knowledge about vaccination and insights into the range of issues to be explored. They included 16 health workers, 7 religious leaders, 5 community leaders, 5 women leaders, 5 health officials and 9 community social mobilisers. These leaders were targeted because they can influence decision-making, community mobilisation for vaccine delivery, enforce regulations and monitor vaccination activities.

### 2.4 Data collection methods

A detailed explanation on the nature of study was given to the participants. Thereafter, informed consent was obtained from each respondent and they were assured of confidentiality. A pretested semi-structured interviewer-administered questionnaire was used to collect data from the caregivers in their various homes at a time convenient to them. The questionnaire sought information on demographic characteristics, previous experience with vaccination, awareness on the development of a malaria vaccine, willingness to accept a malaria vaccine if adopted and information needed to foster acceptability of malaria vaccine. The questionnaire was administered by trained research assistants, and each interview lasted about 10 minutes. The questionnaire was translated into Yoruba, the local language, and back translated into English to ensure adequate translation. Either the English or Yoruba version was used, depending on the respondent’s preference.

### 2.5 Conduct of IDIs

The interviews were conducted among 47 opinion leaders, mostly in their homes and some at their workplaces. Interviews commenced after a detailed explanation of the study purpose was given to the participants. Participants were assured of confidentiality and informed consent was obtained. Permission to use an audio-tape recorder was also obtained. The interviews were carried out using an interview guide in English or Yoruba language, as preferred by the participants. The themes investigated during the interview included: experiences with previous child immunisation, vaccination decision making, malaria vaccine concerns, preferred delivery mechanism and information needs. Trained research assistants conducted the interview. Approximately 20-30 minutes were used per interview.

### 2.6 Data Management

For the survey, SPSS version 15.0 was used for data entry, cleaning and analysis. Summary statistics, such as percentages, means and standard deviations were used for continuous variables and proportions for categorical variables. Chi-square tests were performed to identify factors that were associated with willingness to accept a malaria vaccine. Binary regression analysis was used to determine the strength of association between the factors and willingness to accept malaria vaccine. The level of significance was set at 5%.

For the IDIs, the recorded interview sessions were transcribed and a thematic content analysis was performed on the translated English transcripts [11]. Codes based on themes and sub-themes that were pre-defined to match key objectives of the study and emerging issues from the data were developed. The analysis was carried out independently by both authors, and thereafter their notes were compared. Where there was discrepancy the two investigators revised the analysis together until a consensus was reached. Findings were presented in narratives and supported by quotes.

Ethical approval of the study protocol, including the instruments, was granted by the Joint Ethical Review Committee of the University of Ibadan/University College Hospital, Ibadan (UI/EC/12/0120). Permission to carry out the study was obtained from the Local Government authority and head of households.

## 3 Results

### 3.1 Socio-demographic characteristics of the caregivers

Ages of the respondents ranged from 18 to 45 years, with a mean of 29.8±5.8 years. The majority were married/co-habiting (395, 92.5%) and were mostly females (416, 97.4%). 359 (84.1%) were from monogamous families. Above half (252, 59.0%) were Christians and 175 (41.0%) were Muslims. Two hundred and forty-five respondents (57.4%) had obtained at least a secondary school education and 93 (21.8%) received higher education, whereas 64 (15.0%) had primary school education and few (25, 5.9%) had no form of education. The ethnic composition of the respondents is as follows: 341 (79.9%) were Yoruba, 32 (7.5%) were Hausa and 29 (6.8%) were Igbo, whereas 25 (5.9%) were from other ethnic groups in Nigeria. The occupation the respondents were engaged in included trading (50.8%), artisanship (20.4%), public service (7.7%), housewife (10.1%), teaching (7.5%) and schooling (3.5%).

### 3.2 Caregivers’ experiences with previous child immunizations

Almost all respondents (419, 98.1%) had heard of child immunisation. Of the respondents who had ever heard of immunisation, 409 (95.8%) had had a child vaccinated. The majority of the people who had experienced child vaccination had their children vaccinated with polio vaccine (407, 95.3%), BCG (397, 93.0%) and DPT (350, 82.0%). High fever (205, 48.0%) and abscess at the vaccination site (97, 18.0%) were the major side effects from previous vaccination.

These findings were corroborated by IDI outcomes. When asked if they had ‘ever’ had a child vaccinated before, almost all the respondents mentioned they had ‘ever’ vaccinated at least a child, except one participant who had never vaccinated any child and perceived his children were ‘naturally’ healthy.

“[…] *none of my pikin (children) have ever received vaccination and by the grace of Allah all my children are naturally healthy*”*.* [A male community leader]

The majority of the participants mentioned that their child/ children experienced side effects from vaccination; these included abscess at the injection site, fever or rashes. A few did not report any side effect.

### 3.3 Decision-making and external influences related to child vaccination

The majority of the respondents (364, 85.2%) reported that mothers were generally involved in the decision to give vaccine to their children, and 294 (68.9%) also acknowledged the role of fathers in decision-making for child vaccination. Other people involved in the household decision-making related to child vaccination were mothers-in-law (5.2%), aunts (3.7%), fathers-in-law (1.9%) and uncles (1.4%). Regarding external influences related to child vaccination, 337 (78.9%) of the respondents and less than half (191, 44.7%) reported that health workers at the community level and the mass media, respectively, might encourage and influence child vaccination (Table 1).

**Table 1.** Frequency distribution of decision-makers and external influencers related to childhood vaccination (n=427).

Family decision-makers	Yes (%)	No (%)
Wife (mother)	364 (85.2)	63 (14.8)
Husband (father)	294 (68.9)	133 (31.1)
Mother in-law	22 (5.2)	405 (94.8)
Sisters	16 (3.7)	411 (96.3)
Father in-law	8 (1.9)	419 (98.1)
Brothers	6 (1.4)	421 (98.6)
**External influencers related to child vaccination**		
Health workers at the community level	337 (78.9)	90 (21.1)
Mass media	191 (44.7)	236 (55.3)
Neighbour	74 (17.3)	353 (82.7)
Extended family members	53 (12.4)	374 (87.6)
Religious leaders	51 (11.9)	376 (88.1)
Colleagues	36 (8.4)	391 (91.6)
Village elders	6 (1.4)	421 (98.6)

Note: total percentage exceeded 100% because multiple responses were possible.

During the IDIs, participants were of the opinion that decision-making related to child vaccination was mostly undertaken by mothers and in some cases by both parents. In addition, they mentioned that decision-making depends on the type of family setting. A quote to support this goes thus:

“[…] *Well, we have different family settings. By my own level and by my own disposition I (he) take decision and if am not around the mother (my wife) will take the decision, but it will be a joint decision*”. [A male health Programme Officer]

Some participants noted that the family might also be influenced by a broader network of people that includes colleagues, community leaders, health workers, extended family members, media, friends and pastors.

### 3.4 Awareness of malaria vaccine

The frequency distribution of responses to questions on awareness of a malaria candidate vaccine in development among respondents showed that only few (86, 20.1%) of the respondents had ever heard of the development of a malaria vaccine. Common sources of information on malaria vaccine were clinics (32, 37.0%), family doctors and nurses (15, 17.4%), colleagues (13, 15%) and health seminars (10, 12.0%). Only a few mentioned television (6, 7.0%) and radio (6, 7.0%). Other sources included the work place (2.3%) or pharmacy (2.3%).

Results of IDIs corroborate the survey findings that most of the participants had never heard of a malaria vaccine. Few of the participants had ever heard or were even aware of the vaccine trials currently on-going in several African countries. These participants were those with higher education and health workers, and they mentioned they heard about it through seminars, radio, doctors or workshops.

When participants were asked if they wanted a malaria vaccine to be introduced, all of them welcomed the idea and acknowledged the prospect of a malaria vaccine in the control of the disease. Responses generally reflected the view that a malaria vaccine would bring added health benefits. Many participants, including health officers and community leaders, noted that communities would welcome such a vaccine especially when given orally. Participants commonly held the view that when the vaccine is brought into use it should be safe, readily available and easily accessible. These comments by a community leader and a health programme officer support this view:

“[…] *Government should make sure the vaccine is safe for our children before introducing it. The vaccine should work well; it should be free so that many of our children will be vaccinated*”*.* [A male community opinion leader]

“[…] *The malaria vaccine should be readily available and easily accessible at all the time when it is out for use. The safety of the vaccine should be guaranteed; what are the contents of the vaccine?*” [A female health Programme Officer]

Participants wanted the malaria vaccine to be introduced alongside the existing malaria preventive measures. During the interview, health workers did not see a problem with a new vaccine ‘fitting into’ existing control measures and suggested that vaccine developers should target everyone, including pregnant women and the elderly, and that such a vaccine should be delivered orally. This is supported by a quote from one of the health officers’ responses:

“[…] *Introducing malaria vaccine is a good suggestion; we (people) have been expecting it (malaria vaccine) because these malaria drugs do not work well. For example, like me now I am genotype AA, every month I used to have malaria, if the vaccine can be available to adults like every three months it will be very good. We will use the vaccine with other control measures for good effectiveness. Government and its health officials should be involved in the endorsement of the vaccine. The vaccine should be free and be given orally*”*.*

### 3.5 Respondents’ willingness to accept a malaria vaccine

Most (373, 87%) of the respondents were willing to accept a malaria vaccine, while 54 (13.0%) were unwilling. Reasons stated for unwillingness to accept a malaria vaccine are shown in Table 2. These were: that husbands did not want vaccination (44, 81.0%), it may be expensive (26, 48%) and it may paralyse children (13, 24.1%). All participants who were not willing to accept disagreed with the suggestion that religion forbids children immunisation. Nineteen (35.0%) of the respondents said that if not given orally like polio vaccine it would not be accepted and 9 (16.7%) had fear of injection.

**Table 2.** Respondents’ reasons for not willing to accept malaria vaccine (n=54).

Reason	Agree (%)	Disagree (%)
My husband does not want vaccination	44 (81.0)	10 (19.0)
May be expensive	26 (48.0)	28 (52.0)
If not given orally	19 (35.0)	35 (65.0)
May paralyse children	13 (24.1)	41 (75.9)
Fear of injection	9 (16.7)	45 (83.3)
No money to treat adverse effect	5 (9.3)	49 (90.7)
Immunisation reduces fertility rate of children when they grow up	2 (3.7)	52 (96.3)
Culture forbids child vaccination	1 (1.9)	53 (98.1)
Religion forbids child vaccination	0 (0.00)	54(100.0)

Note: total percentage exceeded 100% because multiple responses were present.

The findings above were corroborated at IDIs, as all the participants, were willing to accept malaria vaccine. A social mobilisation leader in the community said thus:

“[…] *Definitely! I am willing to accept the vaccine; I have been vaccinating my children so I know the importance of vaccination and I will like the children of the community to be vaccinated*”.

The perception of people regarding a malaria vaccine showed that even the person who had never vaccinated a child was willing to accept a malaria vaccine. When the participant who mentioned he has never had a child vaccinated was asked why he was willing to accept, he gave the following response:

“[…] *I will accept malaria vaccine because I see the way malaria is disturbing our children every day especially now that it is rainy season, ‘Malaria ko da rara’ (malaria is not good at all), we want it (malaria) to finish*”*.* [A male community leader]

When participants were asked if they were of the opinion that other people in the country would be willing to accept malaria vaccine, there was a consensus that people will accept the vaccine:

“[…] *Yes, that again depends on how aggressive we are in trying to create awareness; just like any other vaccine that we have been given, polio, DPT and so on, we can just use the mechanism through which awareness was created for these vaccines and the eligible children got vaccinated. The most important thing is to let them know the benefit, why they have to take the vaccine and the level of protection that the vaccine will confer on their children. I think that can solve the problem*”*.* [A male health worker]

Participants gave reasons why people might not accept the vaccine; one major concern was fear of injection and others were safety of the vaccine and cost. However, a few of them suggested that if the vaccine is injectable it should be given alongside with other childhood vaccines and should be given once in a year or probably once in a life time:

“[…] *People may not accept the vaccine because of ignorance, cost of the vaccine, fear of needle and injection abscess, based on past experiences from other childhood vaccination. Even given family planning to adult, made me to understand that most people fear injection and that makes some children not to be vaccinated and not to complete their vaccination*”*.* [A female health worker]

The major motivating factors to accepting malaria vaccine according to participants at IDIs were that if the vaccine is going to be given for free, given orally, and if levels of awareness and sensitisation are high:

“[…] *If government can make announcement (jingles) on radios and televisions this will work on the acceptability. With jingles on radios and televisions and passing on information, people will be able to accept it*”. [A female community leader]

### 3.6 Factors influencing willingness to accept malaria vaccine

*Socio-demographic characteristics and willingness to accept malaria vaccine*

The associations between the socio-demographic factors and willingness to accept malaria vaccine are presented in Table 3. Respondents within the age group 31-45 years (159, 91.4%) constituted a higher proportion of those willing to accept a malaria vaccine compared with those in the age group 18-30 years (214, 84.6%) (*p*=0.04). More Christians (228, 90.5%) reported they would accept the vaccine compared to Muslims (145, 82.9%) (*p*=0.02). Most of the caregivers with higher levels of education (308, 91.1%) were willing to accept malaria vaccine, as compared to caregivers with primary or lower education (65, 73.0%) (*p*<0.001) who were willing to accept. About 319 (88.9%) of those in monogamous families were willing to accept a malaria vaccine compared with those in a polygamous family setting (54, 79.4%) (*p*=0.03). Higher proportions of the Yoruba respondents (312, 91.5%) were willing to accept a malaria vaccine compared with other tribes (46, 85.2%) and Hausa (15, 46.9%), (*p*<0.001).

**Table 3.** Association between socio-demographic characteristics and willingness to accept malaria vaccine.

	Willingness of caregivers to accept a malaria vaccine				
Socio-demographic variables	Yes (%)	No (%)	Total	*Χ^2^*	*p*-value
**Gender**					
Male	10 (90.9)	1 (9.1)	11 (100)	0.129	1.000
Female	363 (87.3)	53 (12.7)	416 (100)		
**Age group (years)**					
18-30	214 (84.6)	39 (15.4)	253 (100)	4.308	0.038*
31-45	159 (91.4)	15 (8.6)	174 (100)		
**Religion**					
Christianity	228 (90.5)	24 (9.5)	252 (100)	5.427	0.02*
Islam	145 (82.9)	30 (17.1)	175(100)		
**Educational status**					
**≤** primary	65 (73.0)	24 (27.0)	89 (100)	20.871	<0.001*
Secondary and above	308 (91.1)	30 (8.9)	338 (100)		
**Marital status**					
Married and co-habiting	347 (87.8)	48 (12.2)	395 (100)		
Single, divorced, widow, separated	26 (81.2)	6 (18.8)	32 (100)	1.167	0.271
**Family type**					
Monogamous	319 (88.9)	40 (11.1)	359 (100)	4.618	0.032*
Polygamous	54 (79.4)	14 (20.6)	68 (100)		
**Tribe**					
Yoruba	312 (91.5)	29 (8.5)	341 (100)		
Hausa	15 (46.9)	17 (53.1)	32 (100)	52.988	<0.0001*
Others	46 (85.2)	8 (14.8)	54 (100)		

* Significant at *p*<0.05

*Decision-making and external influences related to child vaccination and willingness to accept malaria vaccine*

Relationships between respondents’ suggestion of who is involved in decision-making and external influences related to child vaccination and willingness to accept malaria vaccine are shown in Table 4. A higher proportion of the caregivers (328, 90.1%) who agreed that mothers are the key decision-makers for child vaccination, as compared with those who did not agree to this, were willing to accept malaria vaccine (45, 71.4%) (*p*<0.0001). About 266 (91.0%) of the respondents who mentioned that fathers decide on their children vaccine uptake were willing to accept malaria vaccine as compared with respondents who mentioned that fathers are not involved in decision-making (107, 80.5%) (*p*=0.004). Most respondents (312, 92.6%) who mentioned that health workers encourage child vaccination were willing to accept malaria vaccine as compared with those who mentioned that health workers cannot influence their child vaccination (67.8%) (*p*<0.0001).

**Table 4.** Decision-makers and external influencers related to child vaccination and willingness to accept malaria vaccine.

	Willingness of care-givers to accept malaria vaccine				
Decision-making as related to child vaccination	Yes (%)	No (%)	Total (%)	*Χ^2^*	*p*-value
**Husband (father)**					
Yes	266 (90.5)	28 (9.5)	294 (100)	8.331	0.004*
No	107 (80.5)	26 (19.5)	133 (100)		
**Wife (mother)**					
Yes	328 (90.1)	36 (9.9)	364 (100)	16.966	<0.0001*
No	45 (71.4)	18 (28.6)	63 (100)		
**External influencers related to child vaccination Religious leaders**					
Yes	49 (96.1)	2 (3.9)	51 (100)	3.991	0.046*
No	324 (86.2)	52 (13.8)	376 (100)		
**Extended family members**					
Yes	50 (94.3)	3 (5.7)	53 (100)	2.673	0.102
No	323 (86.4)	51 (13.6)	374 (100)		
**Health workers at the community level**					
Yes	312 (92.6)	25 (7.4)	337 (100)	39.55	<0.0001*
No	61 (67.8)	29 (32.2)	90 (100)		
**Mass media**					
Yes	176 (92.1)	15 (7.9)	191 (100)	7.186	0.007*
No	197 (83.5)	39 (16.5)	236 (100)		

* Significant at *p*<0.05

A significant number of the respondents (176, 92.1%) who reported that information about vaccine obtained through mass media could influence child vaccination were willing to accept malaria vaccine compared with those who stated that information from mass media could not influence vaccine uptake (197, 83.5%) (*p*=0.01). There was a significant relationship between religious leaders being key vaccination influencers and willingness to accept a malaria vaccine. A higher proportion (49, 96.1%) of respondents who believed that religious leaders can influence child vaccination were willing to accept malaria vaccine compared with those who did not believe so (324, 86.2%) (*p*=0.046).

*Association between respondents’ awareness and willingness to accept a malaria vaccine*

Caregivers who had ever heard of child immunisation (88.8%) compared with those who had never heard of it were more likely to accept a malaria vaccine (*p*<0.001). Respondents who had ever had a child vaccinated (90.5%) compared with those who never experienced child vaccination were more willing to accept a malaria vaccine (16.7%) (*p*<0.0001).

*Determinants of willingness to accept malaria vaccine*

Variables found to be significant at *p*<0.05 in bivariate analyses were entered into logistic regression models to determine those that predict willingness to accept malaria vaccine. Higher educational status, household decision-maker being the father, community health workers as an external influencer and previous experiences of child vaccination were factors found to be significant predictors of willingness to accept malaria vaccine. With regards to education, the odds of accepting malaria vaccine among respondents with secondary level and higher education were 2.5 times that of respondents with primary education or lower (AOR: 0.39; 95% CI: 0.1-0.85). Respondents who mentioned that fathers decide children vaccine up-takes had three times the odds of accepting malaria vaccine compared with those who mentioned that father did not decide children vaccine uptake (AOR: 0.35; 95% CI: 0.1-0.7). Influence of community health workers and previous experiences of child vaccination were found to predict acceptability of a malaria vaccine. The odds of accepting malaria vaccine were three times higher among those who said health workers could encourage child vaccination compared with those who said health workers cannot do so. (OR: 0.316; 95% CI: 0.142-0.705). The odds of accepting a malaria vaccine were seven times higher among caregivers who had experiences of ever vaccinating a child compared with those who have never had a child vaccinated (OR: 0.146; 95% CI: 0.027-0.799.)

*Information needed and channels to foster acceptability of a malaria vaccine*

Most of the respondents (363, 85.0%) agreed that endorsement of the vaccine by government and health officials, followed closely by involvement of health workers in publicising the vaccination programme (360, 84.3%), providing advanced notice about vaccination days (360, 84.3%), early and comprehensive awareness-creation and health education efforts prior to vaccine introduction (359, 84.1%) were the most important information needed and channels to foster acceptability of a malaria vaccine. More than half of the respondents (264, 61.8%) mentioned the need to involve key religious leaders to assure communities that the vaccine is safe and beneficial for children, and another 262 (61.4%) opined that involvement of influential community members, such as traditional leaders, could help foster the acceptability of malaria vaccine. During IDIs, community health workers, community leaders, religious leaders, mass media, community rally/campaigns, traditional healers and health care providers emerged as some of the trusted sources and key influencers of vaccine uptake within the communities. Participants advised that they should be considered as important channels for health communication. This is illustrated below, using a quote from a female community leader:

“[…] *For any programme that is handed over to the community leader there will be sustainability. It is the community leader that can reach the grassroot, motivate the people, encourage them and monitor the coverage*”.

## 4 Discussion

This study explored the willingness and factors influencing willingness to accept a malaria vaccine if introduced. In an effort to avoid the traditional prescriptive introduction of new interventions including vaccines, this study explored the perspective of the stakeholders including users to determine their preferences, concerns and practices of vaccination. The use of mixed method enriched the data and the findings provided insights into key areas that will influence the acceptability of a malaria vaccine.

### 4.1 Caregivers’ experiences with previous child immunisation

It is encouraging that most of the caregivers have had at least one child immunised. This could be a reflection of the intensified effort at promoting vaccination by way of National Immunisation Days (NIDs), promotion of routine immunisation and the frequency of immunisation campaigns in the country. However, the completeness of immunisation was not elucidated in this study. Complications during previous immunisations, such as high fever and injection abscess, as observed, might affect uptake of a malaria vaccine for children. Studies have shown that complications from previous injections were one of the factors associated with missed opportunities in child vaccination [11]. A report from the US showed that one-sixth of the families studied reported a previous negative immunisation experience related to their children, and this was associated with absence of mothers in the medical home [12].

The candidate malaria vaccine RTS,S will be available in injectable form, and one major question arising from the findings of this study is how this will affect uptake among the caregivers considering that they preferred oral administration. The fact that a few would still accept if it in injectable form could be a rallying point for a campaign; however, the frequency of administration and possibility of administering alongside other childhood vaccines would have to be addressed vehemently.

### 4.2 Decision-making and external influences related to child vaccination

It was observed that mothers tend to decide more frequently on childhood vaccination. Although mothers have the primary responsibility for vaccination decision-making, fathers’ involvement was also acknowledged. Most respondents reported that decision-making for child vaccination takes place within the family unit, generally by one or both parents. This supports the findings, which suggested that mothers were culturally accepted and expected to be key decision-makers for matters related to vaccination services [13]. Research on the ‘Role of Women's Decision Autonomy in the Uptake of Childhood Immunization in Nigeria’ showed that maternal decision-making autonomy is positively associated with the uptake of childhood immunisation services; children whose mothers participated in household decisions had twofold higher odds of being fully immunised compared with children whose mothers did not participate in any household decisions [14]. Furthermore, the result of a study in Ethiopia, which shows that low decision-making capacity of women was strongly associated with lack of immunisation in children and was related to high under-five mortality supported the crucial role of women in vaccine uptake and child care [15].

The positive influence of health workers on child vaccination uptake at the community level, as found in this study, is in contrast to the influence they have on other health services provided such as provision of treatment services and health education at the community level. Health facility attendees often complain of lack of commitment of health facility workers and negative attitudes that hinder utilisation of the facility by the community [16]. This positive influence on vaccination uptake could be explained by their major involvement in the NIDs and campaigns for polio vaccine immunisation, and for which they receive extra stipend. This suggests that health workers might have to be provided with incentives to further the course of introducing a new vaccine in the community. The media stands to be a good complement to the advocacy activities of the health workers, as it was demonstrated in this study to be a channel to encourage and influence child vaccination. The recognition of the religious leaders as trusted influencers of vaccine uptake is very important at this point in time in the country where elimination of polio has been a challenge. The northern part of the country has been a major contributor to this, as their refusal rate is high and this is attributed to misinformation on the true action of the vaccine. [17] The government has had to turn to religious leaders in the northern part of the country to help demystify and mobilise their wards to allow their children to be vaccinated.

### 4.3 Awareness of malaria vaccine

The low awareness of the ongoing development of a malaria vaccine among caregivers in this study is not surprising, as it is still under development and awaits registration; most of the information at this phase of development is directed at scientists, medical professionals and top-level key stakeholders. A study by Kwan *et al.* [18] similarly noted low awareness about other vaccines such as the HPV vaccine among respondents (25.3%) during its development and after its introduction; however, most people wanted more information about it.

Although awareness was low among the interviewees in this study, the majority of the respondents acknowledged the prospect of a malaria vaccine and wanted the vaccine to be introduced along with the existing malaria prevention measures. This finding can be viewed in different ways, as many of the caregivers still lag behind in uptake of the existing malaria preventive measures. For instance, only 28% of under-five children in the country were reported to have slept under a bednet and only 48% of households had one LLIN as required for universal distribution [19]. The uptake of ACT is still below 50% in most studies and use of IPTp is suboptimal. Caregivers might have seen a malaria vaccine as a complementary measure or a measure that is easier to adopt and comply with than existing measures. It is thus important to encourage the optimal use of existing preventive measures prior to the introduction of a vaccine to ensure optimal uptake.

### 4.4 Respondents’ willingness to accept a malaria vaccine

This study found that there was a high level of public interest in a malaria vaccine, despite an overall low awareness of it. The willingness to accept the vaccine was corroborated during the IDIs, and this is in accordance with high willingness observed in other vaccine acceptability studies [20,21]. This study further showed that caregivers who take decisions regarding vaccination of their children and those who reported that health workers at the community level have influenced their decision were those who ever had a child vaccinated. Involvement of these two key stakeholders was also found to be predictors of willingness to accept a malaria vaccine. This is in agreement with the findings by Udezi *et al.* [22], who carried out a study on willingness to pay for three hypothetical malaria vaccines in Nigeria.

The main reason given for why a malaria vaccine might not be accepted is that if there is insufficient information about the efficacy and safety of the vaccine. Some of the people mentioned possible paralysis in the children. Other reasons given were the husband’s refusal, high cost of vaccine, route of administration other than oral, as well as fear of injection. This finding supports the result of Ambe *et al.* [23], who observed that 4% of mothers interviewed in northern Nigeria were not allowed by their husbands to receive immunisation. Participants in this study requested that the vaccine be given for free, suggesting that they might not be able to afford the costs or that they are not willing to pay for the vaccine. This could have borne out of the fact that vaccines for child immunisation in the country are currently free. However, findings of research conducted in Nigeria on the willingness to pay for three hypothetical malaria vaccines suggested that caregivers are willing to pay for the vaccines [22].

In the qualitative part of this study, participants also provided insights into why people might not accept the malaria vaccine and these corroborate the survey findings. Many participants pointed to a lack of understanding of the benefits of vaccination among some people, administration of vaccine by injection (fear of injection), vaccine not given for free and doubt about vaccine safety as potential constrains to vaccine uptake. This is similar to the observation of Saliu *et al.* [24], who found that the cost of a vaccine could preclude adoption, but in variance with the research of Udezi *et al.* [22] on willingness to pay for three hypothetical malaria vaccines in Nigeria. Nicholas *et al.* [20] reported reasons for refusal of pneumococcal vaccine among high-risk patients in America as believing vaccination was unnecessary, fear of injection, fear of vaccine-induced illness and wanting more information regarding the vaccine.

### 4.5 Information needed and channels to foster acceptability of a malaria vaccine

Study participants expressed the need for more information if a new vaccine such as a malaria vaccine were to be introduced. In addition, the importance of early and comprehensive awareness raising, advance notification, accompanied by endorsement of the new vaccine by the national government and its health administrators, involvement of health workers, religious leaders and influential community members were suggested as ways to keep parents and communities informed. The need to address specific concerns about the vaccine and assure the communities that the vaccine is safe and beneficial for children was highlighted in this study and this corroborated the findings of Allison *et al.* [24] and David *et al.* [25].

### 4.6 Factors associated with willingness to accept a malaria vaccine

Those who have health care workers at the community level were more likely to accept a malaria vaccine and this is in agreement with the study of David *et al.* [25], who also found that health care workers at the community level had high influence on the caregivers’ acceptability of a malaria vaccine. The presence of health workers, and provision of efficient health education and promotion services at the community level, therefore, stand to enhance uptake of malaria vaccination in this study area.

It was surprising that the low awareness about malaria vaccine does not influence the willingness to accept it. This was also reported in a study by Aliyu *et al.* [26], which showed no association between HIV awareness and willingness to participate in future HIV vaccine trials across different risk groups in Abuja, Nigeria. It could be that being aware does not translate into knowledge. This is supported by the fact that some of the respondents desired to know more about the vaccine, including the efficacy, safety and formulation. However, there might be other underlying factors that might be responsible for this finding, such as access to health care facilities or religious and cultural beliefs. However, in this study, religion was not identified as a hindrance to acceptance, unlike the experience with polio immunisation in the northern part of the country. Previous related studies [11,27,28] revealed that a high level of education in mothers has a strong association with vaccine uptake, and this was not different in this study. A study carried out in Turkey [29] showed that an increase in level of education of mothers increases the vaccination chance of a child and reduces missed opportunities.

### 4.7 Limitations

The major limitation of this study is that acceptability of a malaria vaccine by caregivers of under-five children in Ibadan North LGA might not be reflective of the acceptability of the entire people of Oyo state and the country at large. The use of one LGA makes the findings not generalisable but provided much-needed information on the willingness to adopt a new vaccine. To address this, a wider or nationwide study might be needed.

## 5 Conclusions

In this study, caregivers were favourably disposed towards the introduction of a vaccine against malaria, despite low awareness of a candidate malaria vaccine. However, they had concerns about the characteristics and formulation of the vaccine, as well as possible adverse effects. As efforts to develop malaria vaccine continue, it will be important to anticipate the health education strategies that will enhance the acceptability of a malaria vaccine and address some of the obstacles to malaria vaccine acceptance highlighted in this study when planning an introduction strategy. These include concerns about vaccine safety, mode of administration and cost. Clarification of the benefits of malaria vaccine might be an important part of a health education strategy. The findings of this study show that parents and other caregivers of children are often influenced by a broad network of people within their communities in making decisions around childhood vaccination. Hence, a communication strategy supporting vaccine introduction should target these people as well as provide needed information and reassurances about immunisation safety. A well-designed communication strategy would be essential to foster a supportive environment for an eventual malaria vaccine introduction.

## References

[1] http://www.who.int/malaria/publications/world_malaria_report_2013/report/en/.

[2] Kristoff J: (2007). Malaria stage-specific vaccine candidates.. Curr. Pharm. Des..

[3] Onyebuchi C. (2014.). National malaria elimination programme reviews progress in Nigeria, a presentation by the Honourable Minister of Health, Nigeria at a 4-day Annual Review Meeting (ARM) of Malaria Programme managers from 17-20 March,. http://www.afro.who.int/en/nigeria/press-materials/item/6406-national-malaria-elimination-programme-reviews-progress-in-nigeria.html.

[4] President’s Malaria Initiative, Nigeria: Malaria Operational Plan FY (2014;). http://www.pmi.gov/docs/malaria-operational-plans/fy14/nigeria_mop_fy14.pdf?sfvrsn=10.

[5] Agnandji ST, Lell B, Soulanoudjingar SS, Fernandes JF (2011). First results of phase 3 trial of RTS,S/AS01 malaria vaccine in African children.. N. Engl. J. Med..

[6] Katahoire RA, Jitta J, Kivumbi G, Murokora D (2008). An assessment of the readiness for introduction of the HPV vaccine in Uganda.. Afr. J. Reprod. Health.

[7] Streefland PH: (2003). Introduction of a HIV vaccine in developing countries: social and cultural dimensions.. Vaccine.

[8] Kabir M: (2005). Knowledge, perception and beliefs about childhood immunization and attitude towards uptake of poliomyelitis immunization in a northern Nigerian village.. Ann. Niger. Med..

[9] Stanton BF: (2004). Assessment of relevant cultural considerations is essential for the success of a vaccine.. J. Health Popul. Nutr..

[10] Program for Appropriate Technology in Health (PATH). (2007).

[11] Abdulraheem IS, Onajole AT, Jimoh AAG, Oladipo RA: (2008). Reasons for incomplete vaccination and factors for missed opportunities among rural Nigerian children.. J. Public Health Epidemiol..

[12] Stockwell MS, Irigoyen M, Martinez RA, Findley S: (2011). How parents' negative experiences at immunization visits affect child immunization status in a community in New York City.. Public Health Rep..

[13] Tanner M, Vlassoff C: (1998). Treatment seeking behaviour for malaria: a typology based on endemicity and gender.. Soc. Sci. Med..

[14] Comfort O: (2012). “Role of Women’s Decision Autonomy in the Uptake of Childhood Immunization in Nigeria” 12^th^ Annual Graduate Research Fair- Presentation,.

[15] Fantahun M, Berhane Y, Byass P (2007). Women's involvement in household decision-making and strengthening social capital-crucial factors for child survival in Ethiopia.. Acta Paediatr..

[16] Ajayi IO, Browne EN, Garshong B, Bateganya F (2008). Feasibility and acceptability of artemisinin-based combination therapy for the home management of malaria in four African sites.. Malar. J..

[17] Jegede AS: (2007). What led to the Nigerian boycott of the polio vaccination campaign?. PLoS Med..

[18] Kwan TT, Chan KK, Yip AM (2009). Acceptability of human papillomavirus vaccination among Chinese women: concerns and implications.. BJOG.

[19] Nigeria Demographic, Health Survey (2013). http://www.measuredhs.com/pubs/pdf/GF15/GF15.pdf.

[20] Daniels NA, Gouveia S, Null D, Gildengorin GL (2006). Acceptance of pneumococcal vaccine under standing orders by race and ethnicity.. J. Natl. Med. Assoc..

[21] Vallabhaneni S, Macalino GE, Reinert SE, Schwartzapfel B (2004). Prisoners’ attitudes toward hepatitis B vaccination.. Prev. Med..

[22] Udezi WA, Usifoh CO, Ihimekpen OO: (2010). Willingness to pay for three hypothetical malaria vaccines in Nigeria.. Clin. Ther..

[23] Ambe JP, Omatara BA, Mandu BM: (2001). Perception, beliefs and practices of mothers in suburban and rural areas towards measles and measles vaccination.. Trop. Doctor.

[24] Bingham A, Janmohamed A, Bartolini R, Creed-Kanashiro HM (2009). An approach to formative research in HPV vaccine introduction planning in low-resource settings.. Open Vaccine J..

[25] Ojakaa DI, Ofware P, Machira YW, Yamo M (2009). Community perceptions of malaria and vaccines in the South Coast and Busia regions of Kenya.. Malar. J..

[26] Aliyu G, Mohammad M, Saidu A, Mondal P (2006). HIV infection awareness and willingness to participate in future HIV vaccine trials across different risk groups in Abuja, Nigeria.. AIDS Care.

[27] Daniel AA, Oladimeji O, Adeyinka FE, Aimakhu C: (2009). Uptake of Childhood Immunization among Mothers of Under-five in Southwestern Nigeria.. Internet J. Epidemiol..

[28] Odusanya OO, Alufohai EF, Meurice FP, Ahonkhai VI: (2006). Determinants of vaccination coverage in rural Nigeria.. BMC Public Health..

[29] Altinkaynak S, Ertekin V, Guraksin A, Kilic A: (2004). Effect of several socio-demographic factors on measles immunization in children of Eastern Turkey.. Public Health.

